# Scanning tunneling microscopy on cleaved Mn_3_Sn(0001) surface

**DOI:** 10.1038/s41598-019-45958-7

**Published:** 2019-07-04

**Authors:** Hung-Hsiang Yang, Chi-Cheng Lee, Yasuo Yoshida, Muhammad Ikhlas, Takahiro Tomita, Agustinus Nugroho, Taisuke Ozaki, Satoru Nakatsuji, Yukio Hasegawa

**Affiliations:** 1 Institute for Solid State Physics, the University of Tokyo, 5-1-5, Kashiwa-no-ha, Kashiwa, Chiba 277-8581 Japan; 2 Department of Physics, Kanazawa University, Kakuma-machi, Kanazawa, 920-1192 Japan; 3 CREST, Japan Science and Technology Agency (JST), 4-1-8 Honcho Kawaguchi, Saitama, 332-0012 Japan

**Keywords:** Electronic properties and materials, Surfaces, interfaces and thin films

## Abstract

We have studied *in-situ* cleaved (0001) surfaces of the magnetic Weyl semimetal Mn_3_Sn by low-temperature scanning tunneling microscopy and spectroscopy (STM/S). It was found that freshly cleaved Mn_3_Sn surfaces are covered with unknown clusters, and the application of voltage pulses in the tunneling condition was needed to achieve atomically flat surfaces. STM topographs taken on the flat terrace show a bulk-terminated 1 × 1 honeycomb lattice with the Sn site brightest. First-principles calculations reveal that the brightest contrast at the Sn site originates from the surrounding surface Mn *d* orbitals. Tunneling spectroscopy performed on the as-cleaved and voltage-pulsed surfaces show a prominent semimetal valley near the Fermi energy.

## Introduction

Mn_3_Sn is a non-collinear antiferromagnetic material that attracts attention in condensed matter physics because of unexpectedly large anomalous Hall effect (AHE) below the Neel temperature of 430 K^1^. Due to a hexagonal crystal structure, the Mn atoms, which have a magnetic moment of ~3 Bohr magneton (*μ*_*B*_) and exhibit antiferromagnetic interaction with neighboring ones, adopt a non-collinear 120-degree spin order^2,3^. Furthermore, a slightly distorted kagome lattice of Mn induces a small net ferromagnetic moment of ~0.002 *μ*_*B*_ perpendicular to the lattice plane^1^. Such a small net moment makes polarity switching of the Hall voltage possible by an application of weak magnetic fields.

Different from ferromagnetic materials, which have an internal magnetic field, the antiferromagnetic AHE of Mn_3_Sn originates from the chiral antiferromagnetic states, which give rise to the presence of Weyl fermions^4–7^. In a recent study, Kuroda *et al*.^4^ evidenced the existence of magnetic Weyl fermions in Mn_3_Sn by angle-resolved photoemission spectroscopy (ARPES). However, surface morphology, atomic termination, and local electronic properties of the cleaved Mn_3_Sn surfaces remain ambiguous. Yin *et al*.^8^ mentioned in their report that the freshly-cleaved Mn_3_Sn shows no clear atomic lattice structure. In search of Weyl fermions of exotic materials, quasiparticle interferences (QPI) measured by STM have been utilized to visualize the Fermi arc^9,10^ as well as the Weyl points^11^. For QPI measurement, it is crucial to obtain atomically flat terraces with resolved lattices.

In this work, we performed STM on *in-situ* cleaved Mn_3_Sn(0001) surfaces and found no atomically flat terraces on as-cleaved surfaces, confirming the previous report^8^. We observed lots of tiny clusters on the cleaved surfaces but found that atomically flat terraces can be locally generated by voltage pulse application to the tunneling junction on the cleaved surfaces. The atomic structure of voltage-pulsed flat surfaces was confirmed as a bulk-terminated one through the careful analysis of the atomic lattices of adjacent terraces and first-principles calculations. To address the electronic structure, we performed tunneling spectroscopy on freshly-cleaved and voltage-pulsed surfaces. Both measurements revealed a semimetallic valley shape and shared a similar minimum near the Fermi level in the tunneling conductance (dI/dV) spectra. The calculated density of states revealed that the minimum is associated with the crossing between valence and conduction bands. The valley was found to be composed of two peaks that arise from Mn d states.

## Methods

**Figure 1:**
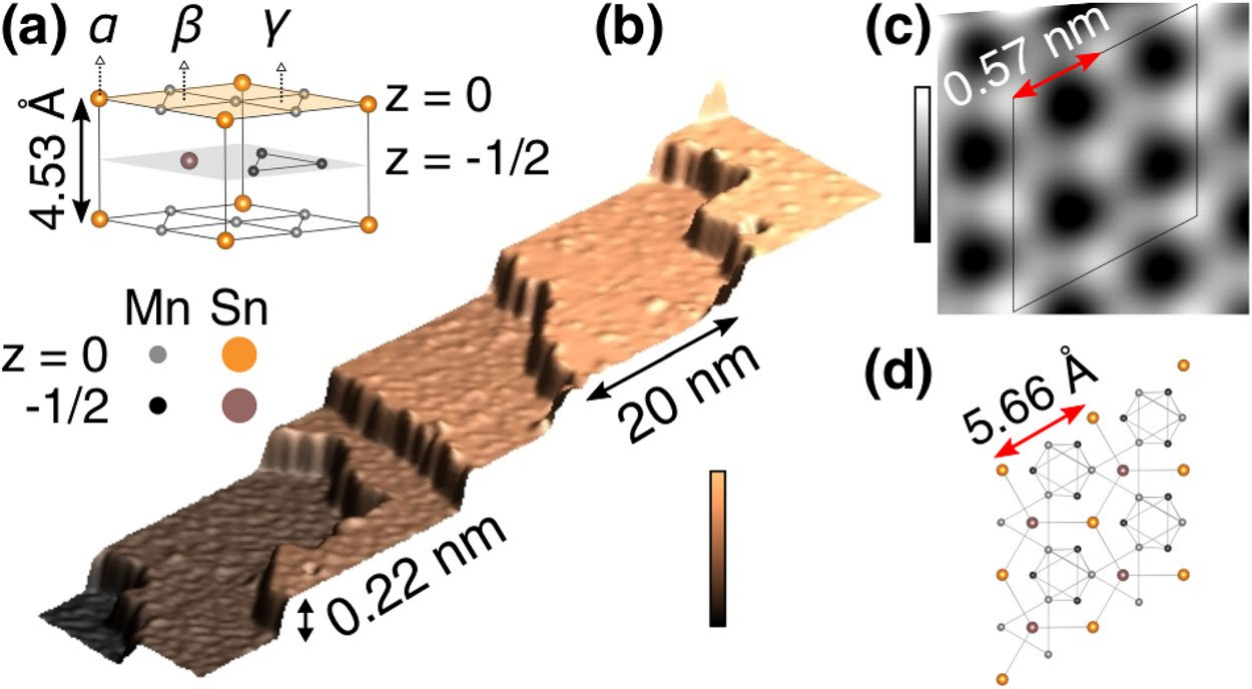
Crystal structure and topography of Mn_3_Sn(0001) surface. (**a**) The unit cell of Mn_3_Sn. Orange (gray) spheres represent Sn (Mn) atoms at z = 0 planes (yellow shaded). Purple (black) spheres represent Sn (Mn) atoms at $z=-\,\frac{1}{2}$ planes (gray shaded). Three surface sites that form a 1 × 1 lattice; *α*, *β*, and γ, are marked. (**b**) 3D-rendered STM image of a voltage-pulsed surface with 20 nm-width terraces separated by a step of 0.22 nm height. (**c**) Typical atomically resolved STM image reveals a honeycomb lattice with the lattice constant of 0.57 nm. (**d**) A 2 × 2 unit cell of the bulk-terminated (0001) surface. Imaging conditions: (**b**) sample bias voltage (*V*_*s*_) −2.0 V, tunneling current (*I*_*t*_): 100 pA, color bar: 0 to 1.31 nm, (**c**) *V*_*s*_ = −50 mV, *I*_*t*_ = 100 nA, color bar: 0 to 35 pm.

Mn_3_Sn has a hexagonal crystal structure whose space group is *P6*_3_*/mmc* with a lattice constant of *a* = *b* = 5.66 Å, *c* = 4.53 Å (Fig. 1(a)). Each *a-b* plane consists of a slightly distorted Mn kagome lattice that shifts by $\frac{1}{3}(\overrightarrow{a}+\overrightarrow{b})$ from *z* = 0 to $z=-\,\frac{1}{2}$ planes. The single crystals we studied were grown by the Bridgman method using a homemade furnace. The samples are Mn-rich with the chemical composition of Mn_3.02_Sn_0.98_. STM measurements were performed with a^3^ He cryostat-based ultrahigh vacuum (UHV) STM (USM-1300S, Unisoku Co. Ltd.) with a Pt_80_Ir_20_ tip and an STM controller (Nanonis SPM control system, SPECS Zurich GmbH). For tunneling spectroscopy, a modulation voltage (frequency: 971 Hz) was applied on the bias voltage and the induced current modulation was measured by a lock-in technique. Mn_3_Sn(0001) surfaces were obtained by cleaving at room temperatures *in situ* under UHV environment whose base pressure <5 × 10^−8^ Pa. All images were processed using Nanotech WSxM^12^.

The first-principles calculations were performed using the OpenMX code, which implements density functional theory (DFT) using the norm-conserving relativistic pseudopotentials and optimized pseudo-atomic basis functions^13–16^. The spin-orbit coupling was incorporated through the j-dependent pseudopotentials^16^. The generalized gradient approximation (GGA) was adopted in our calculations^17^. Three, three, and three optimized radial functions were allocated for the s, p, and d orbitals for each Mn atom, respectively, with a cutoff radius of 6 Bohr, denoted as Mn6.0-s3p3d3. For the Sn atoms, Sn7.0-s2p2d3f1 was adopted. The cutoff energy of 300 Ry was used for numerical integrations and the solution of the Poisson equation. The 7 × 7 × 5 k-point sampling was adopted for the bulk calculation, where two Mn_3_Sn layers were included in the primitive unit cell. The atomic positions were relaxed with spin-orbit coupling until the forces were less than 0.0003 Hartree/Bohr (relaxed unit cell: *a* = *b* = 5.63 Å, *c* = 4.48 Å). For the slab calculations, 14 layers were considered with 4 fully relaxed top layers separated by 15 Å from the next slab in the periodic image. The initial magnetic pattern was set to the inverse triangular spin structure^1^.

## Results and Discussion

Overview STM image (Fig. 1(b)) reveals a step-and-terrace structure whose step height is ~0.22 nm, which is equal to the height of half unit cell. An atomically-resolved STM image, presented in Fig. 1(c) taken with the sample bias voltage (*V*_*s*_) of −50 mV and the tunneling current (*I*_*t*_) of 100 nA, shows a honeycomb lattice whose unit distance is 0.57 nm, agreeing with the lattice constant of Mn_3_Sn (0001) plane (see Fig. 1(c,d)). The tiny and irregular corrugations on the terraces are mainly attributed to the local adsorbates/defects (see Fig. S1 in Supplemental), which are presumbly due to excess Mn atoms from the Mn-rich crystal.

**Figure 2:**
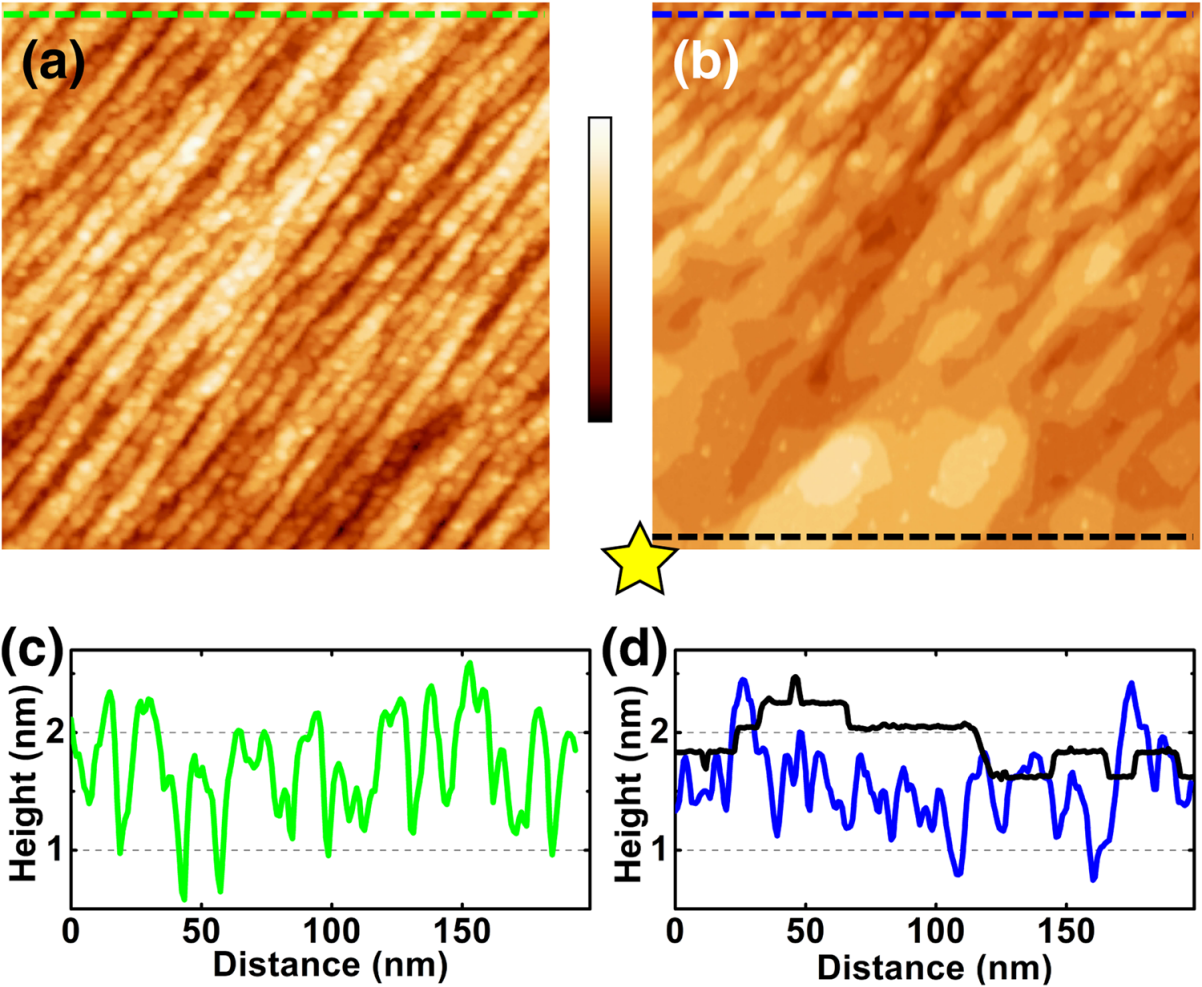
Sample preparation. STM images of (**a**) as-cleaved surface and (**b**) same area as (**a**) after the application of a 10 V pulse at the down-left corner (marked with a star). (**c**) Line profile of the as-cleaved surface (green line in (**a**)). (**d**) Line profiles taken on the voltage pulsed terraces. The black line shows the profile taken in the atomically flat area whereas the blue one was taken in the area 200 nm away from the pulsed site. The lines on which the profiles were measured were drawn in the images with corresponding colors. Imaging conditions: (**a**) *V*_*s*_ = −1.0 V, *I*_*t*_ = 100 pA, (**b**) *V*_*s*_ = −2.0 V, *I*_*t*_ = 100 pA, size: 200 × 200 nm^2^, color bar: 0 to 3.2 nm.

However, the ideal morphological and atomic structures presented in Fig. 1 were not obtained by the cleavage only. Figure 2(a) shows a typical STM image of a Mn_3_Sn surface freshly cleaved under the UHV environment. Whereas the as-cleaved surfaces are overall flat with root-mean-square (RMS) roughness of ~0.5 nm, no atomically flat terraces are found because of the presence of many clusters there. The absence of atomically flat terrace on cleaved Mn_3_Sn surfaces was also reported in a previou report^8^. One of the possible reasons for the cleaved Mn_3_Sn(0001) surface not exhibiting the atomically resolved lattice is a relatively weak intra-layer bonding. The DFT results show that the in-plane Mn-Mn bond (2.697 Å) is longer than the Mn-Mn bond between layers (2.636 Å), which suggests a weaker intra-layer bonding. As a result, during the cleaving, the in-plane bonds are not strong enough to hold the surface intact thus the surface breaks into fragments.

We found that atomically flat area can be formed by the application of voltage pulses (pulse height: *V*_*s*_ = 10 V, duration: 10 ms, with the STM feedback off) to the tip-sample tunneling junction. In the STM image of Fig. 2(b), which was taken in the same area as Fig. 2(a) after the pulse application at the down-left corner of the image (marked with a star), step-and-terrace structure can be seen in the lower part of the image without any clusters on it. Prior to the application of voltage pulses, the tip-sample distance was stabilized with set points ranging from *V*_*s*_ = 0.1 to 1 V and *I*_*t*_ = 50 pA. After the voltage pulse, atomically flat terraces (10 to 50 nm in width) separated by the half-unit-cell steps (~0.22 nm in height) are found (Fig. 2(d)). As shown in the STM images (Fig. 2(a,b)), the flat terrace structure was not formed by just simply removing clusters on it; gradual morphological transition from the clusters to narrow terraces and their coarsening were observed from the upper to the lower part of the image of Fig. 2(b). We thus presume that the formation of the atomically flat terraces is due to local heating by the injection of field-emitted electrons (_>_10 nA) from the tip during the voltage pulse application. The flat-terrace-and-step structure spread in 100 to 200 nm from the pulsed site. The line profile of the surface 200 nm away from the pulsed site (Fig. 2(d), blue) shows peak-to-peak height difference similar to the one of the as-cleaved surface (Fig. 2(c), green). The observation of both cluster-covered and atomically flat areas in the same STM image without any sign of tip change or double/multiple tips confirm that the morphological change induced by the pulse is not a tip artifact. The flat terrace structure can be formed by the 10 V pulse application with a success rate of ~80 %; in the remaining 20 % of the attempts, nothing happened or a large hump (_>_100 nm) was found presumably due to the tip bumping into the sample. Pulses with amplitude between 3 to 10 V have lower successful rate of creating flat terraces. The morphology of the surface remained unchanged with voltage pulses smaller than 3 V.

**Figure 3:**
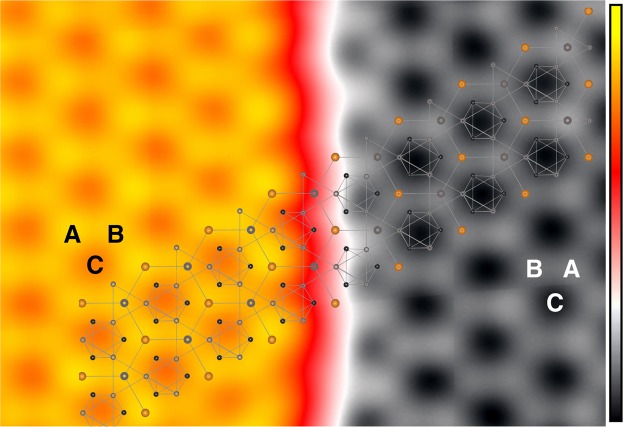
Identification of lattice sites. STM image showing two terraces separated by a step. The brightest spots are marked as A site, the second brightest as B site and the darkest as C site. The A and B sites swap their sites on the adjacent terrace while C site remains the same. (*V*_*s*_ = −50 mV, *I*_*t*_ = 10 nA, 10 × 20 nm^2^, color bar: 0 to 0.29 nm).

In order to reveal which atoms contribute to the lattice contrasts in the STM images taken in the atomically flat area, we carefully investigated the relation of the lattice across a step edge, which is presented in Fig. 3. According to the crystal structure, there are three possible sites that forms a 1 × 1 lattice on a bulk-terminated surface; one is the center of a Mn trimer with a Mn trimer underneath, as marked *γ* in Fig. 1(a). Hereafter we refer the site as Mn_3_-Mn_3_. The other two sites are a Sn atom with a Mn trimer underneath (Sn-Mn_3_ marked as *α*), and the center of a Mn trimer with a Sn atom underneath (Mn_3_-Sn marked as *β*). When two of the three sites are contrasted bright, a honeycomb pattern is observed.

The structural model (Fig. 1(a)) tells us that the Sn-Mn_3_ (*α*) site and the Mn_3_-Sn (*β*) site are swapped when one moves from the topmost plane (*z* = 0) to the next plane ($z=-\,\frac{1}{2}$), whereas the Mn_3_-Mn_3_ (*γ*) sites remain the same. Both terraces in the STM image (Fig. 3) exhibit a honeycomb lattice with the brightest (second brightest) spots marked as A (B). By extending the unit cell from the upper to the lower terrace, one can notice the swapping between A and B. This indicates that either Sn-Mn_3_ or Mn_3_-Sn (*α* or *β* site) is the brightest and the other is second brightest whereas Mn_3_-Mn_3_ (*γ*) sites are dark contrasted.

**Figure 4:**
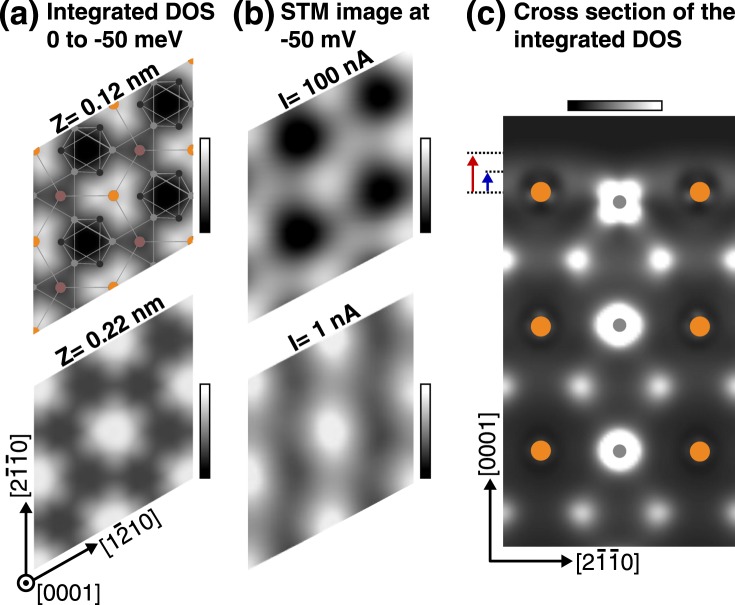
Calculated surface density of states. (**a**) Optimized surface lattice overlays with the DOS integrated from 0 to −50 meV in *a-b* planes. The height of the *a-b* planes is set at 0.12 nm (upper) and 0.22 nm (lower) from the topmost surface Sn atoms. (**b**) STM images taken with *V*_*s*_ = −50 mV and *I*_*t*_ = 100 nA (upper) and *I*_*t*_ = 1 nA (lower). (**c**) Cross-sectional DOS map integrated from 0 to −50 meV in an (01 $\bar{1}$ 0) plane. Gray (orange) circles indicate the center of Mn (Sn) atoms. The arrows indicate the plane cuts for Z = 0.12 nm (blue) and Z = 0.22 nm (red), respectively. Color bar: (**a**) 2.16 to 9.36 $\frac{{10}^{-5}}{eV}$ (upper panel). −2.88 to 8.64 $\frac{{10}^{-6}}{eV}$ (lower panel). (**b**) 0 to 42 pm (upper panel). 0 to 7.4 pm (lowe panel). (**c**) 2.16 to 72.0 $\frac{{10}^{-5}}{eV}$.

A first-principles theoretical calculation provides a key to solve the correspondence between the atomic sites and the contrasts in the honeycomb lattice acquired by STM. The upper panel of Fig. 4(a) shows a density-of-states (DOS) mapping integrated (based on the Tersoff-Hamann scheme) from the Fermi level to −50 meV in an *a-b* plane above the topmost surface atoms by 0.12 nm. An optimized ball-stick model is overlaid on it. The DOS contrast matches well with the STM image taken with *V*_*s*_ = −50 mV and *I*_*t*_ = 100 nA (upper panel of Fig. 4(b)). The lower panel of Fig. 4(a) presents an integrated DOS mapping with the same energy range in an *a-b* plane at 0.22 nm. Regardless of the height from the surface atoms the lowest DOS area is found at theMn_3_-Mn_3_ site whereas the highest (the second highest) locate at Sn-Mn_3_ (Mn_3_-Sn) sites. The STM image with smaller tunneling current (*I*_*t*_ = 1 nA, lower panel of Fig. 4(b)), which corresponds to larger tip-surface distance, shows a structure that qualitatively matches with the DOS mapping at 0.22 nm (lower panel of Fig. 4(a)). The agreement between the DFT and STM images comprehensively confirms that the pulse-cleaned surface is basically a bulk-terminated one in terms of atomic positions. The integrated DOS mapping shows that the Sn atoms correspond to the brightest sites in the observed honeycomb lattice.

Figure 4(c) shows a cross-sectional DOS distribution integrated from 0 to −50 meV in the (01 $$\bar{1}$$ 0) plane crossing a surface Mn atom. The arrows indicate the plane cuts for Z = 0.12 nm (blue) and Z = 0.22 nm (red), respectively. In our calculation, we set z-direction along [0001] and y-direction along [2$$\bar{1}$$$$\bar{1}$$0]. Therefore, Mn d_*yz*_ orbital lies in the plane. At the site of the surface Mn atom, an “x”-shaped state is revealed, which corresponds to the natural appearance of the d _*yz*_ orbital. In contrast, bulk Mn shows spherical shape, implying that the enhanced DOS of the d _*yz*_ orbital is found only at the surface. Due to 3-fold symmetry at the Sn sites, cross sections along [$$\bar{1}$$2$$\bar{1}$$0] and [$$\bar{1}$$$$\bar{1}$$20] exhibit the identical state density. These indicate that the symmetrically-equivalent d_*yz*_ orbitals oriented along the [2$$\bar{1}$$$$\bar{1}$$0], [$$\bar{1}$$2$$\bar{1}$$0] and [$$\bar{1}$$$$\bar{1}$$20] directions contribute to the bright contrast at the Sn sites, where in total 6 d_*yz*_ orbital tails meet. These results indicate that the observed honeycomb state is the consequence of the anticipated imbalanced occupation numbers in the Mn d_*yz*_ and d_*xz*_ orbitals due to the lifted degeneracy (see Fig. S2 in Supplemental) in the kagome structure and explain why the STM image shows a honeycomb lattice.

**Figure 5:**
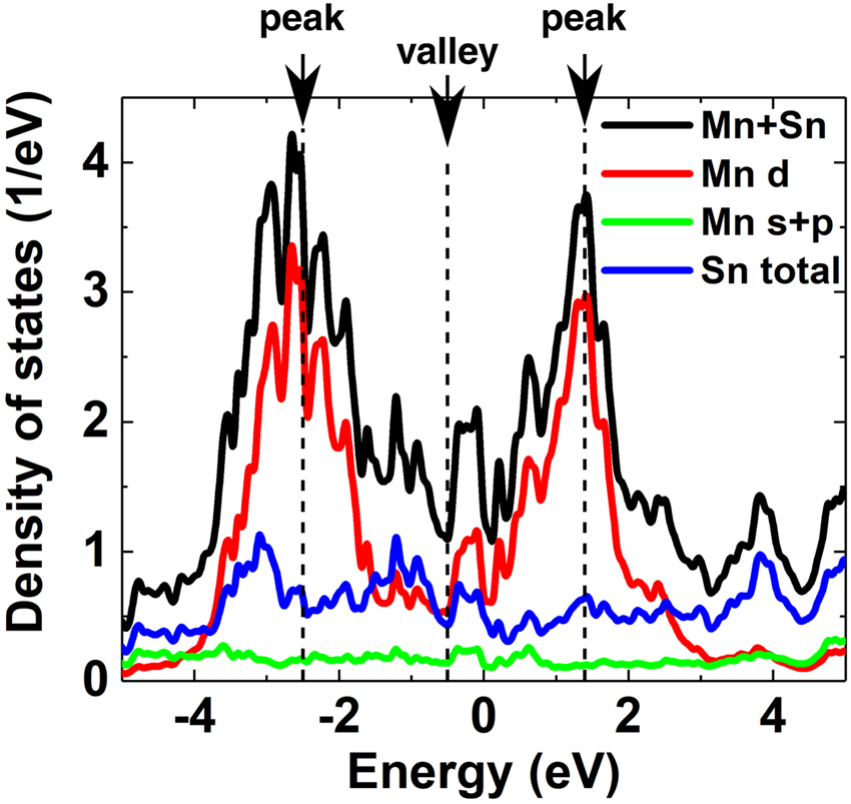
Calculated density of states of surface Mn and Sn atoms with respect to the Fermi level. Black: DOS summed over Mn and Sn atoms; Red: Mn d orbitals; Green: Mn s and p orbitals; Blue: Sn orbitals. The corresponding band structure is presented in Fig. S3(c) in Supplemental. The estimated peak and valley positions without taking into accout the bandwidth renormalization are indicated by arrows based on the overall shape of DOS.

To gain more insight on the DOS contribution from each atom, DOS of relevant orbitals of the surface Mn and Sn atoms are plotted in Fig. 5. Two prominent peaks (indicated by the arrows) can be identified as a contribution from Mn d orbitals: unoccupied spin-minority states and almost fully occupied spin-majority states. Similar results are found in the DOS integrated from the Mn_3_ Sn bulk band structure (see Fig. S3(a,b) in Supplemental). The tail of the spin-minority states contributes more in the vicinity of the Fermi level, where the Coulomb repulsion is not strong enough to give an insulating gap but a semimetal valley (near the Fermi level, pointed out by an arrow). The plot also indicates that the high contrast observed at the Sn sites is not due to the states originating from the Sn atom itself but the d orbitals of the surrounding Mn atoms.

**Figure 6:**
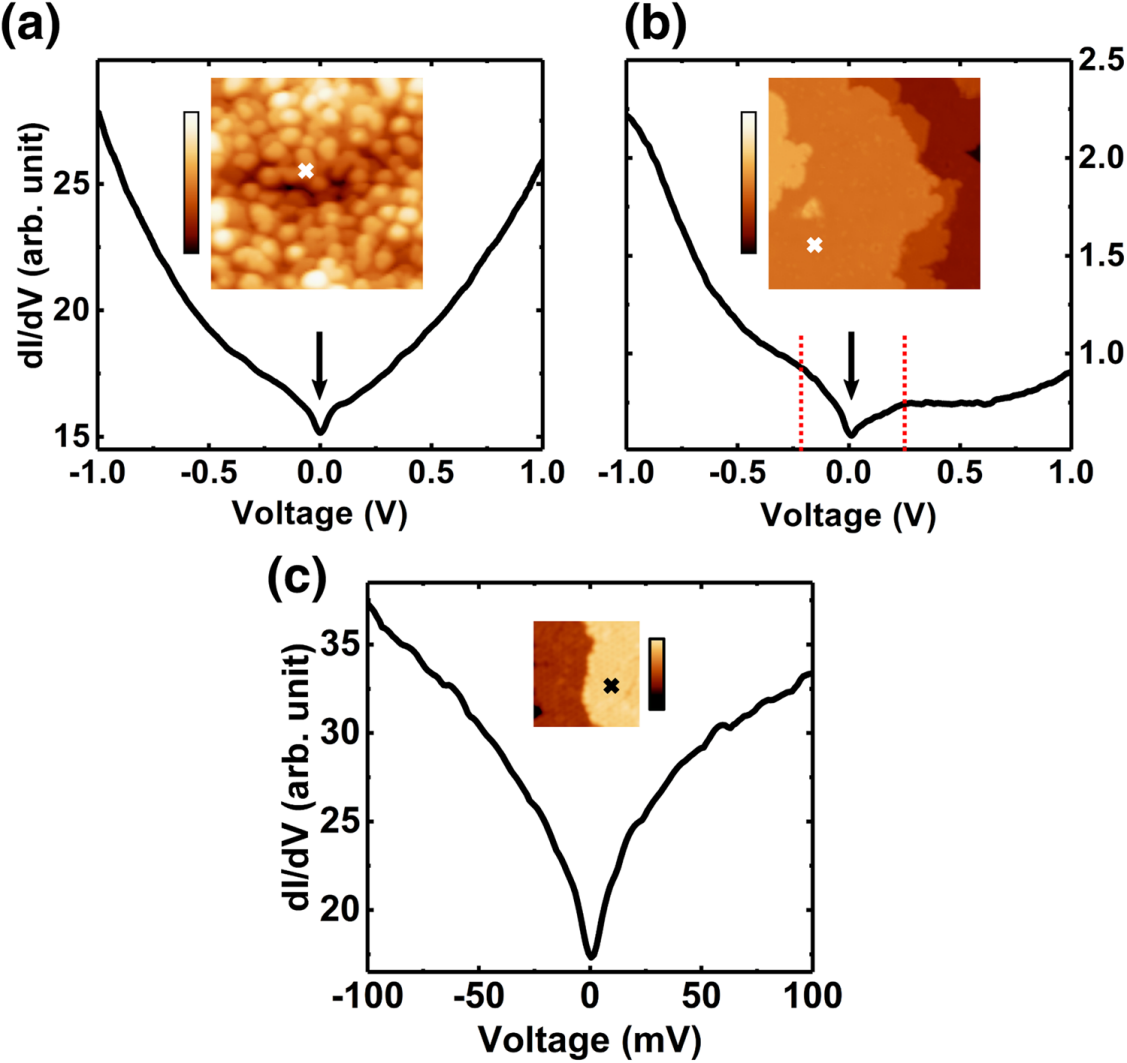
Tunneling spectrum taken on (**a**) as-cleaved surface and (**b**) voltage-pulsed surface. Insets of (**a**,**b**) show the corresponding STM images for the spectroscopic measurements. Crosses indicate the location where the spectrum was taken. Arrows and dotted lines indicate the local minimum near the Fermi level and humps, respectively. (**c**) Zoomed spectrum around the local minimum. The stabilization condition for the spectra (**a**) *V*_*s*_ = −1.0 V, *I*_*t*_ = 1 nA, (**b**) *V*_*s*_ = −2.0 V, *I*_*t*_ = 2 nA, lock-in modulation amplitude: (**a**, **b**) 20 mV, (**c**) 1 mV, imaging conditions for the insets: (**a**) *V*_*s*_ = −1 V, *I*_*t*_ = 200 pA, (**b**) *V*_*s*_ = −50 mV, *I*_*t*_ = 100 pA, (**c**) *V*_*s*_ = −150 mV, *I*_*t*_ = 5 nA, size of the insets: (**a,b**) 20 × 20 nm^2^, (**c**) 10 × 10 nm^2^, Color bar of the insets: (**a,b**) 0 to 0.162 nm. (**c**) 0 to 4.62 Å. Spectra are averaged by 20 curves for (**a**,**b**) repectively, and 120 curves for (**c**). Spatial variations along a line of 10 nm are presented in Fig. S4 in Supplemental.

Figures 6(a) and 6(b) display typical tunneling spectra taken on as-cleaved and voltage-pulsed surfaces, respectively. The inset STM images show the site at which each spectrum was taken and the corresponding surface morphology. A robust local minimum near the Fermi level is identified (marked by arrows) in both tunneling (dI/dV) spectra (see Fig. S4 in Supplemental for their spatial distribution). The energy-zoomed spectrum (Fig. 6(c)) of the voltage-pulsed surface confirms the minimum is located at the Fermi level within an accuracy of 2 meV. Since Mn_3_Sn was recognized as a Weyl semimetal, the plausible origins for the DOS minimum are Weyl points as well as band crossings (We use the term crossing because of the tiny gap of 1.5 meV found in our calculation). There are two pairs of Weyl points around K points near the Fermi level^2^, which might compose the DOS local minimums. However, due to the nature of a semimetal, joints between conduction and valence bands would also construct a local minimum in DOS near the Fermi level. The observed semimetal valley is consistent with the calculated DOS, but for the detailed comparison, theoretical analysis that considers more complete Coulomb correlations is needed.

A renormalization of the bandwidth required for the results of the first-principles calculation to be compared with that measured by angle-resolved photoemission spectroscopy (ARPES) is a signature of strong Coulomb correlations. In Mn_3_Sn, a large renormalization factor, ~5, has been revealed^4^. Such a significant renormalization is also expected for the tunneling spectra. In the spectra taken on the voltage-pulsed surface (Fig. 6(b)), humps can be seen on both sides of the minimum with a peak (marked with dashed lines around −0.2 and +0.25 V). By taking into account the renormalization factor of 5 in the energy scale, the humps correspond to the peaks of the occupied and unoccupied DOS in Fig. 5.

The renormalization also have to be considered in the comparison of the calculated DOS mappings with STM images, which was discussed in Fig. 4. We found that the DOS mappings integrated in two energy ranges; 0 to −50 meV, which corresponds to the applied bias voltage, and 0 to −250 meV, which is wider by a factor of 5 than the bias voltage, exhibit essentially identical features, as shown in Supplemental (Fig. S5). The assignment of the lattice sites discussed in Fig. 4 still holds even with the renormalization.

## Conclusion

In conclusion, we have successfully prepared atomically flat Mn_3_Sn(0001) surface by *in-situ* cleaving of a single crystal sample and subsequent voltage pulsing on the tunneling junction. STM images reveal a honeycomb structure whose brightest sites correspond to the surface Sn atoms. The calculated DOS qualitatively resemble the STM-resolved honeycomb lattice. Cross-sectional DOS shows that the d_*yz*_ orbital of surface Mn atoms contribute to the bright contrast at the Sn sites. In the tunneling spectrum taken on both as-cleaved and voltage-pulsed surfaces, we have observed a local minimum at the Fermi energy. The observed minimum confirms the semimetal nature in Mn_3_Sn, and the origins are presumably from the Weyl points and/or band crossings near the K points. Humps observed in both sides of the Fermi level might be attributed to the Mn d states through the consideration of the bandwidth renormalization.

## Supplementary information

Supplemental
