# Supplementary material for: Scanning tunneling microscopy on cleaved Mn3Sn(0001) surface
Source: Sci Rep. 2019 Jul 4;9:9677. doi: 10.1038/s41598-019-45958-7 (PMC6609646; doi:10.1038/s41598-019-45958-7)
Supplement: Supplementary file 1 — Supplemental [file 41598_2019_45958_MOESM1_ESM.pdf]

Supplemental

Scanning tunneling microscopy on cleaved $Mn_3Sn(0001)$ surface

Hung-Hsiang Yang,[1,*] Chi-Cheng Lee,[1] Yasuo Yoshida,[1,2] Muhammad Ikhlas,[1] Takahiro Tomita,[1] Agustinus Nugroho,[1] Taisuke Ozaki,[1] Satoru Nakatsuji,[1,3] and Yukio Hasegawa[1]

[1] *Institute for Solid State Physics, the University of Tokyo, 5-1-5, Kashiwa-no-ha, Kashiwa, Chiba 277-8581, Japan*

[2] *Department of Physics, Kanazawa University, Kakuma-machi, Kanazawa 920-1192, Japan*

[3] *CREST, Japan Science and Technology Agency (JST), 4-1-8 Honcho Kawaguchi, Saitama 332-0012, Japan*

[*] Email: hh.yang.ntu@gmail.com

DEFECTS ON VOLTAGE-PULSED SURFACE

We observed a small amount of defects/adsorbates on the voltage-pulse applied atomically flat surfaces. Figure S1 shows a zoomed STM image with the tunneling condition of Vs= -10 mV and It= 10 nA, which implies smaller tip-sample distance than the images shown in Figs. 1(b), 2(a) and 2(b). We found triangular-shaped defects locate at A and B site, which are most likely excess Mn atoms because of the Mn-rich crystal. In the case of larger tip-sample distance, which is the case of Fig. 1(b) (Vs= -2 V, It= 100 pA), these defects/adsorbates appear as protrusions larger than the atomic size. Terraces generated by voltage pulses are occasionally accompanied by adsorbates/defects, but defectless area can often be found near step edges (*e.g.* Fig. 3).

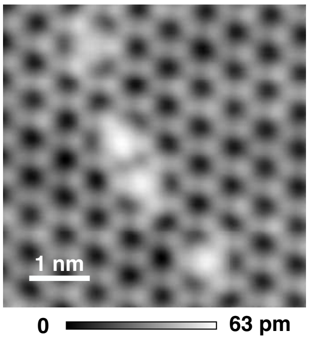

Fig. S 1. Triangular-shaped defects are found at A and B site. STM image taken with the bias voltage of -10 mV and the tunneling current of 10 nA.

PARTIAL DOS OF D-ORBITALS OF MN ATOMS

The degeneracy of Mn d_{xz} and d_{yz} orbitals is lifted due to the broken rotational symmetry, which results in the unbalanced orbital occupation numbers. The presence of the surface further enhances the enegy difference between d_{xz} and d_{yz} orbitals. Figure S2 displays the calculated partial DOS of the surface-Mn atom (left panel) and the bulk-Mn atom (right panel). Near the Fermi level, we found that the surface-Mn atom shows distinct peaks for d_{xy}, d_{xz} and d_{yz} orbitals (marked by arrows). On the contrary, the bulk-Mn atom exhibits similar overall features for d_{xy}, d_{xz} and d_{yz} orbitals. The d_{yz} orbital shows the most pronounced states at the Fermi level and thus give rise to the x-shape DOS distribution at the surface.

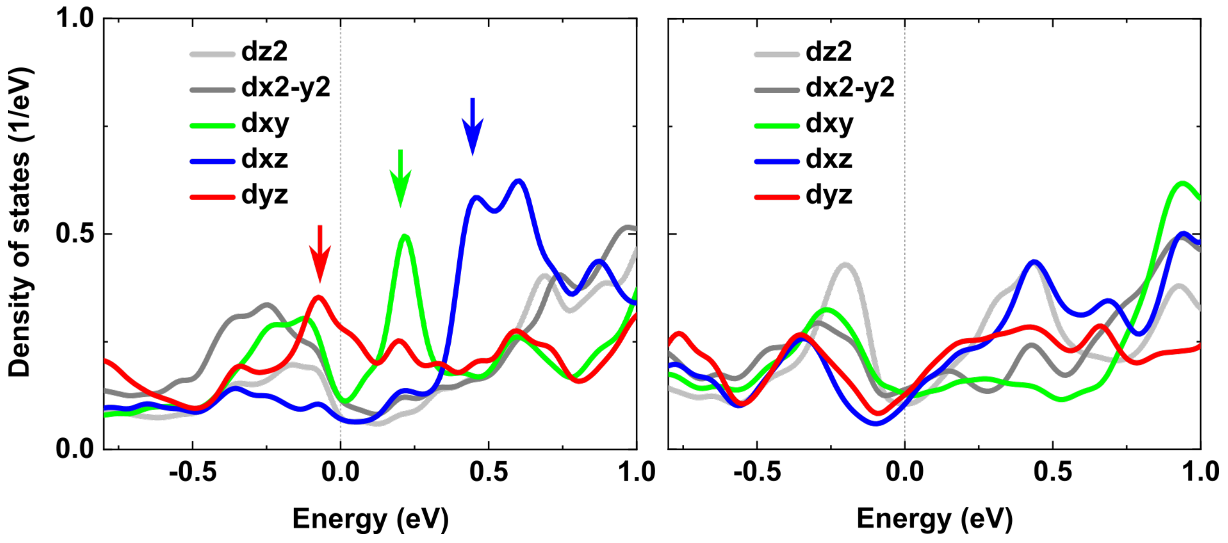

Fig. S 2. (Partial DOS of d-orbitals of the surface-Mn atom (left) and the bulk-Mn atom (right). The arrows indicate the splitting of d_{xy}, d_{xz} and d_{yz} orbitals.

BULK AND SURFACE BAND STRUCTURES

In this study, we performed DFT band calculation for both bulk and surface of Mn_3Sn. For the surface calculation, a 14-layer slab was used. In the bulk band diagram (Fig. S3(a)), a Weyl point is marked by the red arrow. The DOS of the bulk band diagram is dispayed in Fig. S3(b). The surface band diagram as well as the corresponding DOS are presented in Figs. S3(c) and (d), respectively.

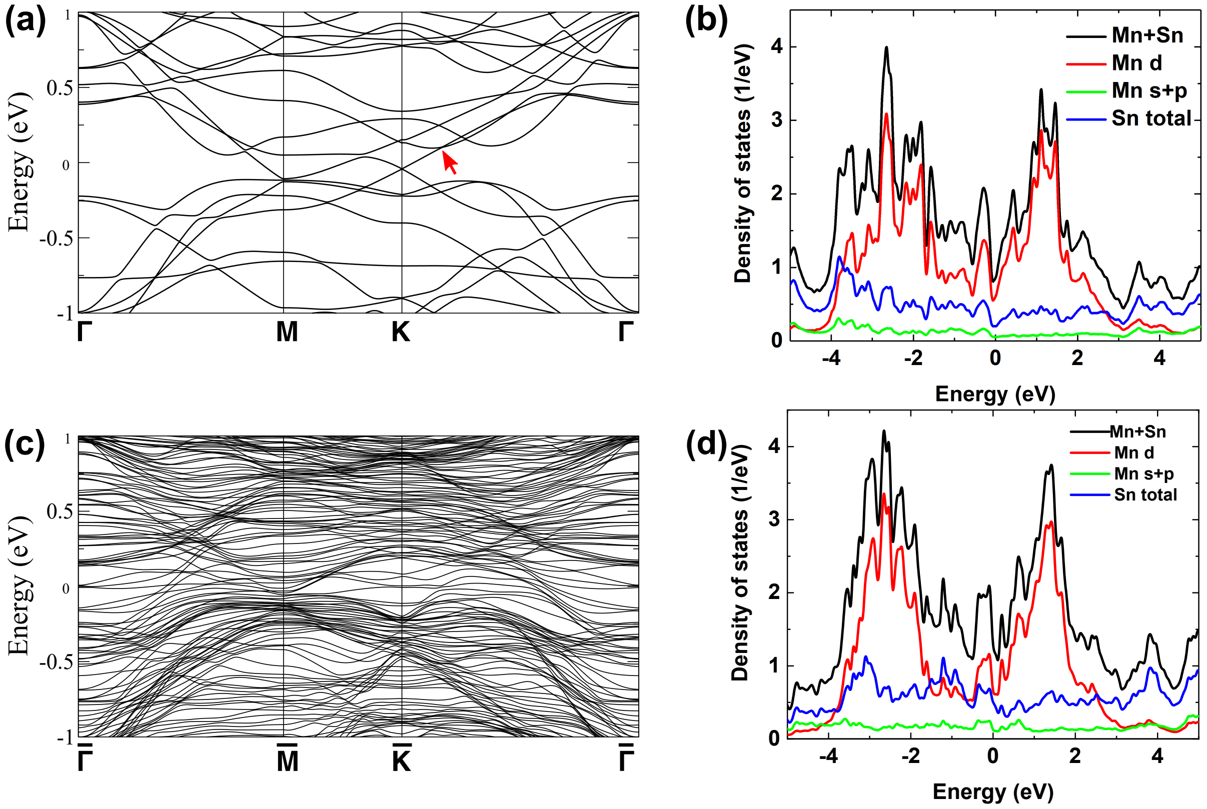

Fig. S 3. (a) calculated band structure of bulk Mn_3Sn and (b) the corresponding DOS. The arrow indicates one of the Weyl points. (c) band structure of Mn_3Sn slab with 14 layers. (d) DOS of the slab calculation.

SPATIAL VARIATION OF THE TUNNELING SPECTRA

The spatial variation of the tunneling spectra taken along a line of 10 nm on freshly-cleaved and voltage-pulsed surfaces are presented in Figs. S4(a) and (b), respectively. We found the local minimum near the Fermi level in both surfaces. The change in the energy position is negligible along the 10 nm line. Minor variations aside from the DOS minimum are presumably due to the influences of the shape of clusters (freshly-cleaved surface) and the adsorbates/defects (voltage-pulsed surface). The spectra taken on both the surfaces share the similar features, and differences in morphology give minor differences in tunneling spectra. Accordingly, regardless of the surface morphology, both surfaces show DOS spectra with the signature of semimetal.

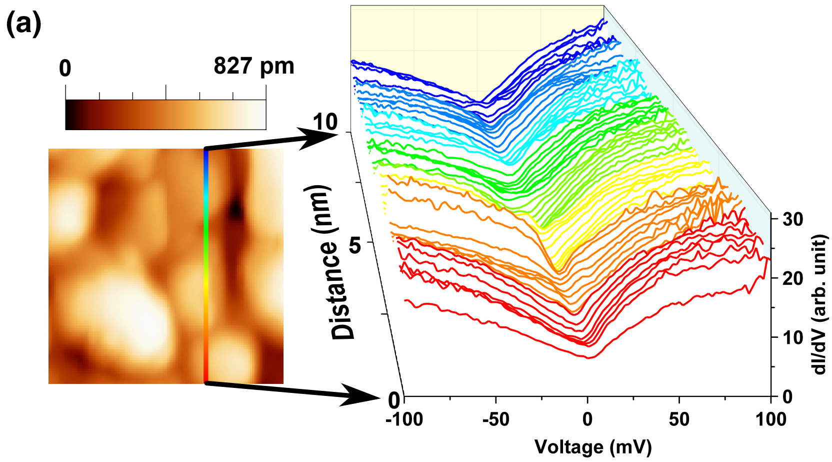

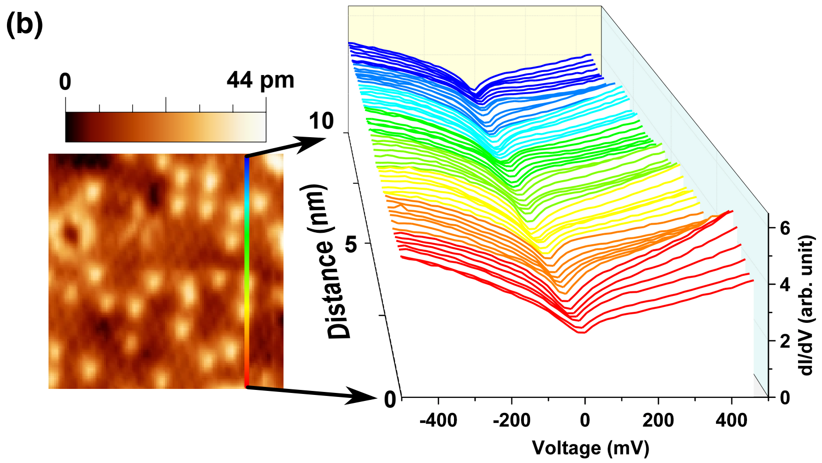

Fig. S 4. Spatial variation of tunneling spectra taken along a line of 10 nm on (a) freshly-cleaved and (b) voltage-pulsed surfaces. Imaging conditions: (a) Vs = 100 mV, It = 100 pA, (b) Vs = -500 mV, It = 1 nA. Stabilization condition for the spectra: (a) Vs = 100 mV, It = 100 pA, (b) Vs = -500 mV, It = 1 nA. Lock-in modulation amplitude: (a) 10 mV, (b) 20 mV.

BANDWIDTH RENORMALIZATION DUE TO STRONG COULOMB CORRELATION

For the calculation of strongly correlated electron bands, it has been known that the renormalization has to be considered in the comparison with the experimentally obtained electronic states, such as those taken by photoemission spectroscopy (PES), and STM. In the case of Mn_3Sn a renormalization factor of 5 was utilized for agreement with the results

of angle-resolved photoemission spectroscopy (ARPES) [S1].

In Figure S5, we compared experimentally obtained STM images taken with the bias voltage of -50 mV with theoretically calculated DOS mappings integrated from the Fermi level to -50 meV, without considering the renormalization, and found the agree well. In Figure S5(c), which is shown below, we obtained the DOS mapping integrated from the Fermi level to -250 meV, 5 times larger than the case of Fig. S5(b). The DOS mapping is, however, basically similar with that of -50 meV, indicating that concerning STM images and DOS mappings the agreement of quite good regardless of whether or not the renormalization is considered.

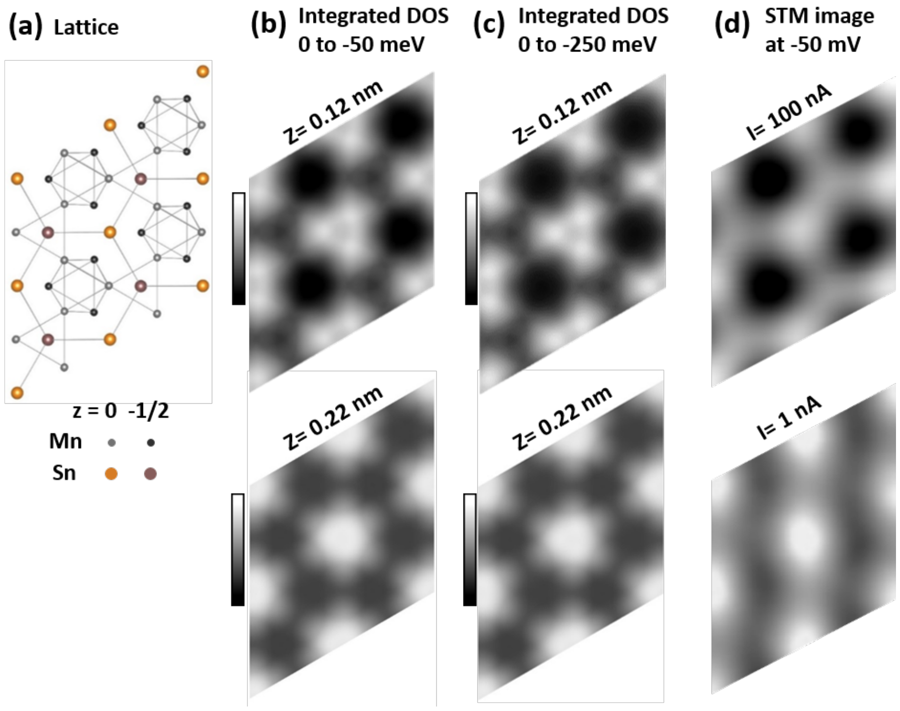

Fig. S 5. Calculated surface density of states. (a) Optimized surface lattice by DFT. (b) Integrated state density in a-b planes between 0 to -50 meV and (c) 0 to -250 meV. The height of the xy planes is set at 0.12 nm (upper) and 0.22 nm (lower) from the topmost surface Sn atoms. (d) STM images taken with the bias voltage of -50 mV and the tunneling current of 100 nA (upper) and 1 nA (lower). Color bar: (b) 2.16 to 9.36 $\frac{10^{-5}}{eV}$ (upper panel). -2.88 to 8.64 $\frac{10^{-6}}{eV}$ (lower panel). (c) 11.0 to 47.6 $\frac{10^{-5}}{eV}$ (upper panel). -1.46 to 4.40 $\frac{10^{-5}}{eV}$ (lower panel).

REFERECES

[S1] K. Kuroda, et al. Nature Material, 16, 1090 (2017).
